# Gestational weight gain and gestational diabetes among Emirati and Arab women in the United Arab Emirates: results from the MISC cohort

**DOI:** 10.1186/s12884-019-2621-z

**Published:** 2019-12-03

**Authors:** Mona Hashim, Hadia Radwan, Hayder Hasan, Reyad Shaker Obaid, Hessa Al Ghazal, Marwa Al Hilali, Rana Rayess, Noor Chehayber, Hamid Jan Jan Mohamed, Farah Naja

**Affiliations:** 1 Department of Clinical Nutrition and Dietetics, College of Health Sciences, Research Institute of Medical and Health Sciences (RIMHS), University of Sharjah, Sharjah, United Arab Emirates; 2 Family Health Promotion Center, Sharjah, United Arab Emirates; 3 Al Qassimi Hospital, Ministry of Health and Prevention, Sharjah, United Arab Emirates; 4 Nutrition and Dietetics Program, Universiti Sains Malaysia, Kelantan, Malaysia; 5 Department of Nutrition and Food Sciences, American University of Beirut, Beirut, Lebanon

**Keywords:** Gestational weight gain, Gestational diabetes, Pre-pregnancy BMI, UAE

## Abstract

**Background:**

Nutritional status of women during pregnancy has been considered an important prognostic indicator of pregnancy outcomes.

**Objectives:**

To investigate the pattern of gestational weight gain (GWG) and gestational diabetes mellitus (GDM) and their risk factors among a cohort of Emirati and Arab women residing in the United Arab Emirates (UAE). A secondary objective was to investigate pre-pregnancy body mass index (BMI) and its socio-demographic correlates among study participants.

**Methods:**

Data of 256 pregnant women participating in the cohort study, the *Mother-Infant Study Cohort (MISC)* were used in this study. Healthy pregnant mothers with no history of chronic diseases were interviewed during their third trimester in different hospitals in UAE. Data were collected using interviewer-administered multi-component questionnaires addressing maternal sociodemographic and lifestyle characteristics. Maternal weight, weight gain, and GDM were recorded from the hospital medical records.

**Results:**

Among the study participants, 71.1% had inadequate GWG: 31.6% insufficient and 39.5% excessive GWG. 19.1% reported having GDM and more than half of the participants (59.4%) had a pre-pregnancy BMI ≥ 25 kg/m^2^. The findings of the multiple multinomial logistic regression showed that multiparous women had decreased odds of excessive gain as compared to primiparous [odds ratio (OR): 0.17; 95% CI: 0.05–0.54]. Furthermore, women with a pre-pregnancy BMI ≥ 25 kg/m^2^ had increased odds of excessive gain (OR: 2.23; 95%CI: 1.00–5.10) as compared to those with pre-pregnancy BMI < 25 kg/m^2^. Similarly, women who had a pre-pregnancy BMI ≥ 25 kg/m^2^ were at higher risk of having GDM (OR: 2.37; 95%CI: 1.10–5.12). As for the associations of women’s characteristics with pre-pregnancy BMI, age and regular breakfast consumption level were significant predictors of higher pre-pregnancy BMI.

**Conclusions:**

This study revealed alarming prevalence rates of inadequate, mainly excessive, GWG and GDM among the MISC participants. Pre-pregnancy BMI was found a risk factor for both of these conditions (GWG and GDM). In addition, age and regular breakfast consumption were significant determinants of pre-pregnancy BMI. Healthcare providers are encouraged to counsel pregnant women to maintain normal body weight before and throughout pregnancy by advocating healthy eating and increased physical activity in order to reduce the risk of excessive weight gain and its associated complications.

## Background

Pregnancy is among the most critical periods of development in the human lifespan, whereby exposures during this period are postulated to have lifelong implications on the health of women as well as their offspring. The Developmental Origins of Health and Disease (DoHaD) hypothesis proposed a link between prenatal, perinatal and early postnatal exposure to certain environmental, dietary, and lifestyle factors and subsequent development of obesity and non-communicable diseases [1]. Among lifestyle factors, gestational weight gain (GWG) has been identified as a major predictor of obstetric, neonatal outcomes as well as health later in life [2].

The influence of GWG on pregnancy complications and outcome depend on pre-pregnancy BMI, hence the Institute of Medicine (IOM) has set the guidelines for GWG according to pre-pregnancy BMI [2]. Previous studies have shown that adequate pregnancy weight gain is crucial for optimal outcomes for both mothers and infants [3, 4]. More specifically, restricted GWG lower than the recommendations were found to be associated with stillbirth, infant death, and child neurocognitive development and behavior [5]. On the other hand, excessive GWG during early pregnancy was associated with an increased risk of gestational hypertension and preeclampsia, caesarean delivery, macrosomia, and post-partum weight retention after delivery [6–8]. Furthermore, excessive GWG was found to exacerbate the generational impact of obesity, whereby women who exceed the recommended weight gain during pregnancy are more likely to retain weight post-partum and to enter the next pregnancy with a higher BMI and deliver heavier babies, who have higher odds to become overweight or obese adults later in life [9].

A common complication associated with excessive weight gain during pregnancy is gestational diabetes mellitus (GDM), characterized by glucose intolerance, of variable degrees, with an onset first recognized during pregnancy [10]. A plethora of literature documented the adverse health implications of GDM for both mother and child. Although glucose homeostasis normalises shortly after delivery, a woman with GDM remains at a higher risk to develop type 2 diabetes mellitus (T2DM) later in life [11], increasing her predisposition to cardiovascular, renal and retinal diseases. In fact, the results of a meta-analysis showed that the relative risk to develop T2DM among women with GDM was 7.7, 95%CI (4.79–11.51) compared to those who had a normoglycemic pregnancy [12]. With regards to the health of the newborn, GDM was found to increase the risk of fetal macrosomia by 15–45%, if the baby was born to a mother with GDM compared to a mother with normal glucose homeostatis [13]. A baby who is born with a weight greater than the 90th percentile or above 4000 g is typically considered a microsomic baby [14]. Macrosomia is associated with numerous fetal compalications such as shoulder dystocia, perinatal asphyxia, hyperinsulinemia, neonatal jaundice and neonatal morbidity [11, 13].

In light of the significant health implications of GWG and GDM on the health of the mother and child, it is critical to understand their prevalence and determinants among various populations in order to develop evidence-based interventions and inform public health policies. Most of the current evidence on GWG and GDM stems from studies conducted in Western countries making it difficult to compare or generalize the findings to other parts of the world. Few studies have evaluated patterns of weight gain, GDM correlates and pre-pregnancy BMI in countries where malnutrition and poor weight gain, as well as maternal obesity, are known to coexist [15–18].

The UAE is an economically fast-growing country in population size and per-capita income [19]. The rapid modernization and economic growth provoked a shift in diet and lifestyle factors which triggered a marked increase in the prevalence of overweight and obesity and metabolic abnormalities [19, 20] **.** A recent report by Yusufali et al., showed that 41.9 and 19.6% of women in the UAE were overweight and obese respectively [21].

To the best of our knowledge, there is little known about the patterns of GWG and GDM in the UAE. The main objective of this study was to investigate the pattern of GWG and GDM and their risk factors among a cohort of Emirati and Arab women residing in the UAE. A secondary objective was to investigate pre-pregnancy BMI and its sociodemographic correlates among study participants.

## Methodology

Data for this study were drawn from the *Mother-Infant Study Cohort (MISC)*, a prospective cohort study that included 256 pregnant women from the UAE. Detailed descriptions of the study methods and the recruitment were previously published [22]. Pregnant women in their third trimester were selected using a convenient sampling approach from three main public governmental hospitals, and seven Primary Health Care (PHC) clinics and Mother and Child Centers (MCH) in the Emirates of Sharjah, Dubai, and Ajman. Subjects’ recruitment took place during the period from December 2015 to December 2017. In the MISC, data collection is planned at six time points (third trimester, at delivery, 2, 6, 12 and 24 months postpartum). For the purpose of this study, data pertinent to the first time point (third trimester) were used.

Ethical approvals were obtained from all ethics boards overseeing conduct of research in the Emirates of Dubai, Ajman, and Sharjah including Research and Ethics Committee at the University of Sharjah (REC/14/01/1505), Al Qassimi Clinical Research Centre Ethical Research Committee (REC Reference Number: 21512015 ± 03), Ministry of Health Ethical Research Committee (R02), and Dubai Health Authority (DSREC-0/2016). Before enrollment in the study, participating women provided written informed consent.

The inclusion criteria were Emirati and Arab expatriate pregnant women within their 3rd trimester (27–42 weeks of gestation), aged 19 to 40 years old; with singleton pregnancy, free of chronic diseases such as (diabetes, hypertension, kidney disease, and cancer), and not planning to leave the UAE for the duration of the study. Exclusion criteria were: pregnant women with multiple pregnancies and those who were diagnosed as high-risk pregnancy or had a history of chronic diseases.

In the clinics/hospitals, data was collected using interviewer-administered multi-component questionnaires addressing maternal sociodemographic and lifestyle characteristics such as age (in years), nationality (Emirati or Arab), occupation (employed versus housewife), education (intermediate or less, high school/technical diploma and university), parity (primiparous versus multiparous), income, parity, daily breakfast consumption (daily versus breakfast skippers), and physical activity. The latter was assessed using the Pregnancy Physical Activity Questionnaire (PPAQ) [23], whereby total physical activity was calculated by weighting each type of activity by its energy requirements defined in The metabolic equivalent of task (MET) (multiples of the resting metabolic rate for an activity multiplied by the minutes performed). Based on METS-min per week, three categories of physical activity were assigned, including low, moderate, and high intensity.

### Gestational diabetes mellitus (GDM)

The clinical diagnosis of GDM was obtained from the clinical record. Pregnant women participating in this study were screened for GDM during their 24–28 weeks of gestation using the National Institute for Health and Care Excellence (NICE) Diabetes in Pregnancy criteria [24].

### Maternal pre-pregnancy body mass index (BMI)

Maternal pre-pregnancy BMI referred to BMI before pregnancy. The latter was calculated using height and pre-pregnancy weight. Mother height was obtained during the visit using standard protocol and was measured to the nearest 0.1 cm (cm) using Seca 220 Telescopic Measuring Rod for Column Scales. (As for the weight before pregnancy, it was extracted from the medical record. In this record, women were asked to report their last weight before pregnancy which was recorded during the first antenatal visit. BMI was calculated as weight (in kg) divided by square height (in meter). Then the pre-pregnancy BMI was categorized according to the World Health Organization (WHO) classification [25]: BMI less than 18.5 kg/m^2^ as underweight, BMI 18.5 to 24.9 kg/m^2^ as normal weight, BMI 25.0 to 29.9 kg/m^2^ as overweight, and BMI 30.0 kg/m^2^ or greater as obese.

### Gestational weight Gain

GWG was calculated as the difference between the recorded pre-pregnancy weight of the mother and the last weight measured before delivery which was derived from the medical records. Further, GWG was categorized as having gained insufficient, adequate, or excessive weight relative to their pre-pregnancy BMI according to the IOM guidelines. Accordingly, adequate GWG was a function of pre pregnancy BMI. Inadequate weight gain was defined as the gestational weight gain either above or below the IOM recommendations These guidelines recommend that women who are underweight should gain 12.5 to 18 kg, women of normal weight should gain 11.5 to 16 kg, women who are overweight should gain 7 to 11.5 kg, and obese women should gain 5 to 9 kg over the course of their pregnancy [2].

### Statistical analysis

Participants’ characteristics were presented as means ± standard deviation (SD) and proportions for continuous and categorical variables respectively (*n* = 256). Simple and multiple multinomial logistic regressions were used to examine the effect of participants’ characteristics on GWG. In these regression analyses, GWG was the dependent variable (with normal GWG as the reference category) and participants’ characteristics as the independent variables. For the association between these characteristics and GDM, simple and multiple logistic regression analyses were used with GDM as the dependent variable. Similarly, simple and multiple logistic regressions were applied for the association between participants’ characteristics and pre-pregnancy BMI ≥ 25 kg/m^2^. In all analyses, variables with a *p* value of < 0.25 in simple regressions were included in the multiple regression models in addition to age. A *p* -value < 0.05 was used to indicate statistical significance. The Statistical Package for Social Sciences (SPSS) version 22 (IBM Corp. Released 2013. IBM SPSS Statistics for Windows, Version 22.0. IBM Corp: Armonk, NY, USA) was used for data cleaning, management, and analyses.

## Results

Out of a total of 420 women who were approached to participate in the MISC, 256 women completed visit 1 and were included in the analysis for this study (response rate 61%).

Descriptive characteristics of the study participants are presented in Table 1. The average age of study participants was 30.5 ± 6.0 years, and the majority (53.5%) were ≥ 30 years. The study sample included more Arab women then Emirati (59% versus 41%). Only 13.7% of the participants have an intermediate or lower education level, while 54.7% had a high school or diploma and 31.6% were holders of a university degree. The majority of women were housewives (82.4%) and the monthly family income exceeded 10,000 AED (UAE currency; 1 US $ = 3.67 AED) for 53.4% of women and only 9.3% had an income of > 5000 AED for 53.4% of women and only 9.3% fell below 5000 AED. Almost three in four women participated in this study were multiparous (76.6%). A considerable proportion of women had low-intensity physical activity (64.8%) and consumed breakfast daily (69.9%). With regards to pre-pregnancy BMI, more than half of the participants (59.4%) had a BMI ≥25 kg/m^2^. Among study participants, 71.1% had inadequate GWG: 31.6% insufficient and 39.5% excessive GWG. Furthermore, 19.1% reported having GDM (Table 1).

**Table 1:** Descriptive characteristics of pregnant women participating in this study (*n* = 256)

	Total^a^
Mother’s Age (years)	30.5 ± 6.0
18–24.9	57 (22.3)
25–29.9	62 (24.2)
≥ 30	137 (53.5)
Nationality	
Emirati	105 (41.0)
Arab^b^	151 (59.0)
Education	
Intermediate or less	35 (13.7)
High School/ Technical Diploma	140 (54.7)
University	81 (31.6)
Employment Status	
Employee	45 (17.6)
Housewife	211 (82.4)
Family Monthly Income (AED)	
< 5000	18 (9.3)
5000-10,000	72 (37.3)
> 10,000	103 (53.4)
Parity	
Primiparous	60 (23.4)
Multiparous	196 (76.6)
Physical Activity (METs)	
Low-Intensity Activity	166 (64.8)
Moderate / High-Intensity Activity	90 (35.2)
Daily Breakfast Consumption	
No	77 (30.1)
Yes	179 (69.9)
Pre-pregnancy BMI (kg/m^2^)^c^	27.3 ± 6.2
BMI < 25	104 (40.6)
BMI ≥ 25	152 (59.4)
Gestational weight gain (GWG)^d^	
Insufficient	81 (31.6)
Adequate	74 (28.9)
Excessive	101 (39.5)
Gestational diabetes mellitus (GDM)	
No	207 (80.9)
Yes	49 (19.1)

^a^Values in the table are presented as n(%) for categorical variables and mean (SD) for continuous variables

^b^it includes mothers of different Arab Nationalities such as: Syrian, Jordanian, Sudanese, Palestinian, Lebanese, and Algerians etc. …

^c^According to WHO classification 2007; ^d^ According to IOM classification 2009

The associations of various characteristics of study participants with GWG were examined using simple and multiple multinomial logistic regression (Table 2). For these associations, the adequate GWG was used as reference category. The results of the simple regression showed that both parity and pre-pregnancy BMI were significantly associated with GWG. Multiparous women were less likely to have excessive GWG (*p*<0.05). Women with a pre-pregnancy BMI ≥25 kg/m^2^ had higher odds of gaining excessive weight during their pregnancy (*p*<0.05) (Table 2). These findings were confirmed by those of the multiple multinomial logistic regression, after adjustment for age. More specifically multiparous women were 83% less likely to have excessive weight gain as compared to primiparous (OR: 0.17; 95% CI: 0.05–0.54). Furthermore, women with a pre-pregnancy BMI ≥ 25 kg/m^2^ had 2.23 times the odds of excessive GWG as compared to those < 25 kg/m^2^ (OR: 2.23; 95%CI: 1.00–5.10) (Data not shown).

**Table 2:** Simple multinomial regression analysis for the association of subjects’ characteristics with gestational weight gain (GWG)

	Insufficient	Excessive
	OR (95%CI)	OR (95%CI)
Mother’s Age (years)		
18–24.9	Ref	Ref
25–29.9	0.64 (0.26,1.58)	0.64 (0.26,1.57)
≥ 30	0.68 (0.31,1.53)	0.95 (0.43,2.07)
Nationality		
Emirati	Ref	Ref
Arab	0.95 (0.501,1.79)	1.15 (0.62,2.11)
Education		
Intermediate or less	Ref	Ref
High School/ Technical Diploma	1.80 (0.65,5.00)	0.96 (0.41,2.26)
University	2.57 (0.86,7.65)	1.16 (0.46,3.00)
Employment Status		
Employee	Ref	Ref
Housewife	0.80 (0.36,1.79)	1.22 (0.54,2.75)
Family Monthly Income (AED)		
< 5000	Ref	Ref
5000-10,000	0.40 (0.10,1.71)	0.79 (0.18,3.54)
> 10,000	0.28 (0.07,1.15)	0.64 (0.15,2.74)
Parity		
Primiparous	Ref	Ref
Multiparous	0.53 (0.24,1.20)	0.43 (0.20,0.94)
Physical Activity (METs)		
Low-Intensity Activity	Ref	Ref
Moderate /High Intensity Activity	0.69 (0.36,1.34)	0.74 (0.40,1.39)
Daily Breakfast Consumption		
No	Ref	Ref
Yes	0.905 (0.45,1.77)	1.05 (0.54,2.03)
Pre-pregnancy BMI^a^		
BMI < 25	Ref	Ref
BMI ≥ 25	0.615 (0.33,1.16)	2.88 (1.502,5.534)
Gestational Diabetes		
No	Ref	Ref
Yes	1.30 (0.56,3.05)	1.69 (0.77,3.73)

^a^The reference category is ‘Adequate’

**Fig. 1:**
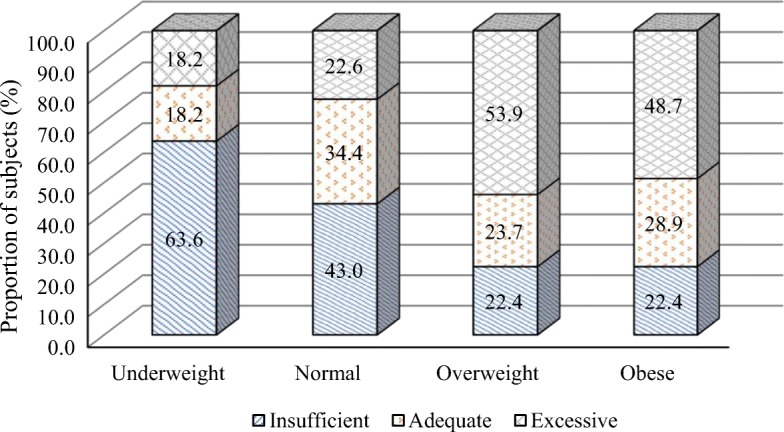
Comparison of weight gain across different body mass index categories

Analysis Of Variance (ANOVA) showed no significant difference among the absolute GWG (expressed in Kg) for underweight (<18.5 kg/m^2^), normal (18.5–24.9 kg/m^2^), overweight (25–29.9 kg/m^2^) and obese (≥ 30 kg/m^2^) (13.45 ± 6.43, 12.53 ± 6.09, 12.50 ± 7.78, 10.34 ± 8.28 respectively, *p* > 0.05). The distribution of the various categories of GWG (insufficient, adequate and excessive), according to pre-pregnancy BMI were depicted in Fig. 1. While the proportions of insufficient GWG decreased across increasing pre-pregnancy BMI, those of excessive GWG increased. Proportions of women with insufficient GWG were 63.6, 43, 22.4, and 22.4%, while the proportions of women with excessive GWG were 18.2, 22.6, 53.9 and 48.7% among women who were underweight, normal, overweight and obese before pregnancy respectively (Fig. 1). It is important to note that the highest proportion of women with adequate GWG was among those with a normal pre-pregnancy BMI (34.4% among women with a normal pre-pregnancy BMI versus 18.2, 23.7 and 28.9% who were underweight, overweight and obese before pregnancy, respectively).

As for the associations of subjects’ characteristics with GDM, the results of the simple logistic regression indicated that older age (≥30 years), daily breakfast intake, and prepregnancy BMI (≥25 kg/m^2^) were associated with higher odds of GDM (*p* <0.05) (Table 3). After age-adjustment, only pre-pregnancy BMI was found to be associated with GDM, whereby, women with a pre-pregnancy BMI ≥25 kg/m^2^ had 2.37 times the odds of having GDM, as compared to those with pre-pregnancy BMI < 25 kg/m^2^ (OR: 2.37; 95%CI: 1.10–5.12) (Data not shown).

**Table 3:** Simple logistic regression analysis for the associations of subjects’ characteristics with Gestational Diabetes

	OR (95%CI)
Mother’s Age (years)	
18–24.9	Ref
25–29.9	1.44 (0.48,4.35)
≥ 30	2.81 (1.11,7.11)
Nationality	
Emirati	Ref
Arab	0.91 (0.49,1.71)
Education	
Intermediate or less	Ref
High School/ Technical Diploma	1.37 (0.49,3.86)
University	1.71 (0.58,5.06)
Employment Status	
Employee	Ref
Housewife	0.59 (0.28,1.24)
Family Monthly Income (AED)	
< 5000	Ref
5000-10,000	1.55 (0.40,5.98)
> 10,000	1.28 (0.34,4.84)
Parity	
Primiparous	Ref
Multiparous	1.07 (0.51,2.25)
Physical Activity (METs)	
Low-Intensity Activity	Ref
Moderate /High Intensity Activity	0.98 (0.51,1.88)
Daily Breakfast Consumption	
No	Ref
Yes	2.56 (1.14,5.77)
Pre-pregnancy BMI^a^	
BMI < 25	Ref
BMI ≥ 25	2.82 (1.37,5.82)

^a^The reference category is: No

Simple and multiple logistic regression analyses were used to investigate the associations of subjects’ characteristics with pre-pregnancy BMI. The results of the simple regression showed that age, education level, parity as well as daily breakfast intake were significantly associated with pre-pregnancy BMI (Table 4). After adjustment, only age and breakfast consumption maintained significant associations. Compared to women aged 18–24.9 years, those 30 years of age or older had significantly higher odds of having a pre-pregnancy BMI ≥25 kg/m^2^ (OR: 4.75; 95%CI: 1.85–12.20). Furthermore, subjects who consumed breakfast regularly were 2.21 more likely to have a pre-pregnancy BMI ≥25 kg/m^2^, as compared to those who did not consume breakfast regularly (OR: 2.21, 95%CI: 1.10–4.44) (Data not shown).

**Table 4:** Simple logistic regression analysis for the association of study characteristics with pre-pregnancy BMI (BMI ≥ 25)

	OR (95%CI)
Mother’s Age (years)	
18–24.9	Ref
25–29.9	1.85 (0.89,3.87)
≥ 30	5.19 (2.67,10.08)
Nationality	
Emirati	Ref
Arab	1.02 (0.62,1.70)
Education	
Intermediate or less	Ref
High School/ Technical Diploma	38(.16,0.90)
University	0.90 (0.16,0.96)
Employment Status	
Employee	Ref
Housewife	1.50 (0.79,2.87)
Family Monthly Income (AED)	
< 5000	Ref
5000-10,000	2.08 (0.72,6.00)
> 10,000	2.48 (0.89,6.91)
Parity	
Primiparous	Ref
Multiparous	2.15 (1.20,3.87)
Physical Activity (METs)	
Low-Intensity Activity	Ref
Moderate /High Intensity Activity	1.04 (0.62,1.76)
Daily Breakfast Consumption	
No	Ref
Yes	1.94 (1.13,3.34)

## Discussion

This is the first study to report results from the MISC cohort, one of a few mother and child cohorts in the Middle East and North Africa (MENA). The study investigated GWG, GDM and their correlates among the MISC participants, a cohort of Emirati and Arab women residing in the UAE, and identified the sociodemographic correlates of pre-pregnancy BMI. The main findings of this study included alarming prevalence of inadequate GWG (insufficient and excessive) and of GDM. Furthermore, while parity and pre-pregnancy BMI were found to be predictors of excessive GWG, only pre-pregnancy BMI was associated with higher odds of GDM. Pre-pregnancy overweight and obesity were correlated with a higher mother’s age and regular breakfast consumption.

A main finding of this study was the significant prevalence of insufficient and excessive GWG that was observed among the participants, with only 30% of participants falling within the adequate GWG as per the IOM guidelines. More specifically, 32and 39% had insufficient and excessive GWG, respectively. These rates are within the range of those reported by a recent systematic review and meta-analysis aimed to examine GWG and which included 23 studies, with a total sample size of 1309 pregnant women. The results of this review indicated that 23 and 47% had insufficient and excessive GWG, respectively [8] . The high prevalence of inadequate GWG and specifically the excessive GWG found in this study is of public health concern, especially in the light of mounting evidence for its impact not only on the birth outcome but also on disease risk later in life [26, 27]. A more direct implication of the excessive GWG among pregnant women is the higher risk of these women to retain weight and become overweight and obese, especially following more than one pregnancy. In fact, women who gained excessive weight during pregnancy were 3.2 times more likely to retain their weight postpartum, and twice as likely to retain at least 5 kg of weight post-pregnancy [28]. The direct effect of excessive GWG on obesity is critical, especially in the context of the UAE, where obesity prevalence rates are soaring [29]. These findings call for a concerted action among various concerned health authorities for proper prenatal nutritional counseling and early interventions to target pregnant who are at risk of inadequate GWG. Therefore, identifying the context specific correlates of inadequate GWG is important to control GWG.

In this study, excessive GWG was found to be associated with parity and pre-pregnancy BMI. With regards to parity, primiparous women were more likely to gain excessive weight compared to multiparous participants. Findings of this study were in accordance with other investigations that reported primiparous women gaining more pregnancy weight or were more likely to exceed GWG recommendations than multiparous women [30–32]. It is arguable that women with more children spend less time at rest and are more likely to be active looking after existing children as compared to women with no children [33]. As for the association between pre-pregnancy BMI and GWG, the results of this study indicated that being overweight or obese prior to pregnancy significantly increased the odds of excessive GWG. Consistent with our findings, Weisman et al., in a study among 103 pregnant women in the USA, reported that being overweight or obese substantially increases the odds of gaining excessive weight [34]. Furthermore, Deputy et al. showed that pre-pregnant overweight and obese women were about 2 and 3 times respectively more likely to have excessive weight gains above the IOM recommendations [35]. Begum et al., indicated that higher pre-pregnancy BMI is a significant predictor of excessive weight gain during pregnancy; 80% of the overweight or obese pregnant women gained weight more than the recommended value [36]. A possible explanation for this phenomena is that overweight or obese mothers may have a high energy diet and low levels of physical activity during their pregnancy which may lead them to gain excessive weight [37, 38]. In light of the study findings regarding the correlates of inadequate GWG, specific nutrition and lifestyle counselling interventions are encouraged to target primiparous as well as overweight and obese women during antenatal care visits to prevent further increase in weight during pregnancy. It is important to note, however, that the absolute GWG values did not vary significantly among the various categories of pre pregnancy BMI. The association between GWG classification and pre pregnancy BMI could have been confounded by the different targets/recommendations of weight gain for each BMI category.

In addition to GWG, this study aimed to examine GDM and its correlates among the MISC participants. The findings of this study showed an alarmingly high prevalence of GDM (19%). Previous studies in the UAE reported that GDM prevalence varied between 7.9 and 24.9% [39]. A few studies reported similar and even higher prevalence rates. For instance, in Vietnam and in Singapore, the prevalence of GDM was 20.06 and 18.93%, respectively [40]. Furthermore, in a cohort study in Saudi Arabia, a higher GDM percentage was reported among 2354 participants (24.2%) [41]. However these prevalence rates are higher than those reported by other Gulf countries, (4.2% in Oman, 16.3% in Qatar, and 10.1% in Bahrain) [42], and also higher than the median prevalence of GDM estimate obtained by a recent review in the MENA (12.9%) [43]. Moreover, lower GDM rates were reported in other parts of the world; by some Asian countries where the prevalence of GDM among Korean mothers was 4.5, and 6.2% among Chinese [44]. Similarly, in Europe, lower GDM occurrence was reported from Epifane, a French birth cohort, where 7.7% of the women had GDM [45]. In Italy, a prospective study that included 14,109 women, GDM was diagnosed in 360 women (2.6%) [46]. As such, the high prevalence of GDM in the UAE raises major public health concern, especially given the mounting evidence for its association with maternal and neonatal complications during pregnancy as well as adverse health outcomes for both mothers and their newborns [47]. GDM is considered to reflect the underlying T2DM epidemic since many of the women with a history of GDM may be imposed to a sevenfold increased risk of T2DM in later life [12]. This adds to the growing burden of diabetes risk among the population in the UAE. The latter has one of the world’s highest prevalence rates of T2DM of 18.7% and is expected to reach 21.4% by 2030 [47].

The high prevalence of GDM reported in this study as well as other reports from the UAE, and its significant health sequalae underscores the need to investigate the determinant of GDM. The results of the multinomial logistic regression in this study showed a significant association between pre-pregnancy BMI and the incidence risk of GDM. The participants with a pre-pregnancy BMI ≥25 kg/m^2^ were over two times more likely to have GDM, as compared to those with pre-pregnancy BMI < 25 kg/m^2^. A meta-analysis based on 31 cohort studies with 364,668 subjects found that women with obesity had higher odds of developing GDM compared to women of normal weight [OR of 3.76(3.31–4.28)]. Other studies also reported similar findings [48].

Another study reported similar findings, whereby mothers with pre-pregnancy overweight or obesity had a 2.19 fold the risk of developing GDM [44]. Together these aforementioned studies confirmed that pregestational obesity is an independent risk factor for GDM [49–52]. In a previous study in the UAE, a higher risk of GDM increased almost 4 times (OR 3.75, 95% CI, 1.83–7.69, *p* = 0.001) in the morbidly obese group in comparison to controls [53]. Maternal obesity is consistently pointed out as a major and modifiable risk factor for GDM [54]. Since GDM and obesity are frequently comorbid conditions, it is well established that women who were diagnosed with GDM during pregnancy, their pre-pregnancy BMI was shown to increase the risk of prediabetes and diabetes later in life [7, 55]. It has been estimated that compared to women with a normal BMI, the risk of pregnancy diabetes is 2 and 4 times higher in overweight and obese women, respectively [50].

The association between a higher pre-pregnancy BMI and GDM might be explained by the fact that obese women, due to greater fat deposition, have lower insulin sensitivity as compared normal weight women [56]. Prevention of GDM is considered a key strategy for breaking the intergenerational cycle of obesity and diabetes [57]. Thus, strategies aiming at preventing obesity in young women and keeping appropriate preconception weight in pre-pregnant women are essential for the prevention of GDM.

Given that Pre-pregnancy BMI has emerged as a strong predictor of GWG and GDM, it was addressed as a secondary objective for this study inorder to identify the sociodemographic correlates of pre-pregnancy BMI among the participants. More than half of the participants in our study were either overweight or obese before pregnancy. This is consistent with other investigators [15, 17]. While other studies reported lower prevalence rates of maternal pre-pregnancy BMI overweight and obesity among their pregnant participants, where about one-third of women were either overweight or obese [58–60].

In this study, maternal age and breakfast consumption were found to be significantly associated with pre-pregnancy BMI. Pre-pregnancy BMI was significantly higher among women who were older and who consumed breakfast regularly. With regard to age, many studies had reported that mothers who were older in age were more likely to be obese before pregnancy [6, 61]. For instance, Boudet-Berquier et al. found that women aged 30-34 years, were more likely to have obesity before pregnancy than to be of normal weight [45]. A possible explanation could lay in the fact that as the mother increases in age, their physical activity and energy expenditure levels decrease [62, 63]. Furthermore, an older age is most likely to be accompanied by multiple pregnancies and child birth which could result in weight retention and development of overweight and obesity. Very few studies examined the association between breakfast consumption and pre pregnancy BMI. Contrary to our findings, a study among Korean pregnant women showed that regular breakfast consumption was more prevalent among women with a normal or underweight pre pregnancy BMI as compared to those with overweight or obese pre pregnancy BMI [64]. In this context, it is suggested that energy density and nutrient composition of the breakfast could be an important confounder for the association between the frequency of consumption and pre pregnancy BMI. More specifically, a breakfast high in simple as opposed to complex carbohydrates tend to be associated with a higher BMI, whereas a breakfast richer in protein, fruits and vegetables could be associated with lower BMI [65]. It is therefore recommended that future studies examining the association between breakfast consumption and pre pregnancy BMI take into consideration energy and nutrient composition of the breakfast in addition to its frequency.

### Strengths and limitations

To our knowledge, this is the first study which investigated GWG, GDM and its correlates and the sociodemographic factors related to pre-pregnancy BMI in the UAE. Moreover, it was the first to use the international IOM recommendations to examine GWG in the UAE. Besides the publication that described the protocol of the MISC [22], this manuscript is the first to report on the results of this cohort. The latter being one of few cohorts in the region investigating early metabolic programming of lifelong health to facilitate identifying at-risk women and developing tailored interventions.

We are aware of important limitations of this study. First the small sample size could have led to underpowered analyses and a higher risk of type II error, especially in relation to GDM and its determinants. Such a sample size also restricted a more detailed classification of pre pregnancy BMI. Second, the pre-pregnancy BMI, GWG, as well as GDM, were extracted from the participants’ records. Although in the clinics and health centers where recruitment took place, standards techniques and guidelines were implemented in obtaining weight and diagnosing GD, it is inevitable that random errors could have occurred. Third, the information collected regarding socio-demographic and lifestyle characteristics was based on subjects’ reporting and hence may have been subject to recall error. That said, the field workers were trained to implement standard interviewing techniques with minimal leading questions in order to minimize any interviewer bias or social desirability bias. Fourth, the low response rate observed in this study could potentially lead to selection bias. Lastly, it is recommended that future longitudinal studies investigating the determinants of GDM take into consideration important variables such as family history of diabetes and a history of gestational diabetes in previous pregnancies.

## Conclusions

This study revealed alarming prevalence rates of inadequate, mainly excessive, GWG and GDM among the MISC participants. Pre-pregnancy BMI was found to be a common denominator in the etiology of both excessive GWG and GDM. In addition, age and education were significant determinants of pre-pregnancy BMI.

As such, women of reproductive age with a high BMI should be given additional attention in targeted pre-conceptional and inter pregnancy interventions in order to prevent GWG and GDM which will prepare them to start the next pregnancy with a healthier BMI. Hence, healthcare specialists are encouraged not only to focus on fetus health during the clinic visit of pregnant mothers but also should pay more attention to maternal health. They should advise them on appropriate weight gain during pregnancy by promoting healthy diet and physical activity in order to prevent GDM and postpartum weight retention and decrease obesity related risks in subsequent pregnancies.

## Data Availability

The datasets used and/or analysed during the current study are available from the corresponding author on reasonable request.

## Abbreviations

ANOVA: Analysis Of Variance
BMI: Body Mass Index
CI: Confidence Interval
Cm: Centimeters
DoHaD: Developmental Origins of Health and Disease
GDM: Gestational Diabetes Mellitus
GWG: Gestational Weight gain
IOM: Institute of Medicine
MCH: Mother and Child Centers
MENA: Middle East and North Africa
MET: The Metabolic Equivalent of Task
MISC: Mother-Infant Study Cohort
NICE: National Institute for Health and Care Excellence
OR: Odds Ratio
PHC: Primary Health Care
PPAQ: Pregnancy Physical Activity Questionnaire
REC: Research and Ethics Committee
SD: Standard Deviation
SPSS: Statistical Package for Social Sciences
T2DM: Type 2 Diabetes Mellitus
UAE: United Arab Emirates
WHO: World Health Organization
